# The prognostic value of selective neck dissection in early-stage major salivary gland carcinoma: a population-based analysis

**DOI:** 10.3389/fonc.2024.1347339

**Published:** 2024-05-22

**Authors:** Meiyu An, Jiaxin Zuo, Fang Yuan, Ping Xiong

**Affiliations:** Department of Ultrasound, Shanghai Ninth People’s Hospital, School of Medicine, Shanghai Jiao Tong University, Shanghai, China

**Keywords:** SEER, major salivary glands malignancy, selective neck dissection, Cox proportional-hazards model, prognosis

## Abstract

**Objective:**

This population-based study aims to assess the survival benefits of selective neck dissection (SND) compared to neck observation in patients with T1/T2N0M0 major salivary gland malignancy (MSGC).

**Methods:**

We conducted a retrospective review of T1/T2N0M0 MSGC patients who underwent primary tumor surgical extirpation with or without elective neck dissection in the Surveillance, Epidemiology, and End Results database (SEER) from 2004-2015. The impact of SND and clinical variables on overall survival (OS) and disease-specific survival (DSS) was evaluated using Univariate and Multivariate Cox proportional hazards regression models. Kaplan-Meier survival curves were generated, and survival rates were assessed via the log-rank test.

**Results:**

Of 3778 post-operative T1-T2N0M0 MSGC patients, 2305 underwent elective neck dissection, while 1473 did not. Median follow-up was 106 months. Univariate and Multivariate analysis identified SND as a prognostic factor for OS in all the study population. After stratified analysis, we found that in the poorly high-grade (differentiated and undifferentiated) patients, the survival showed a significant OS and DSS benefit after receiving SND compared with the neck observations [HR for OS (95%CI): 0.571(0.446-0.731), P<0.001] and [HR for DSS (95%CI): 0.564(0.385-0.826), P=0.003], other than in the well differentiated or moderately differentiated subgroup. Especially, when the pathological is squamous cell carcinoma, the results show that the people underwent SND had better prognosis, not only in OS [HR (95%CI): 0.532(0.322-0.876), P=0.013], but also in DSS [HR (95%CI): 0.330(0.136-0.797), P=0.014]. The multivariate analysis also yielded encouraging results, compared with neck observation, receiving SND bought about a significant independent OS (adjusted HR, 0.555; 95% CI, 0.328-0.941; P=0.029) and DSS (adjusted HR, 0.349; 95% CI, 0.142-0.858; P=0.022) advantage in high grade squamous cell carcinoma MSGC patients. The Kaplan-Meier survival curves also demonstrated that adjusted SND still had significantly better OS(P=0.029) and DSS(P=0.022) than the observation group in patients with high-grade squamous cell carcinoma of MSGC.

**Conclusion:**

Poorly differentiated and undifferentiated T1/T2N0M0 major salivary gland malignancy treated with selective neck dissection demonstrated superior survival compared to neck observation, especially in the pathological subtype of squamous cell carcinoma. These findings suggest the potential benefits of multimodal therapy for appropriately selected patients, emphasizing significant clinical implications.

## 1 Introduction

Major salivary gland cancers (SGCs) constitute 5% of head and neck cancers (1). These tumors arise primarily from the parotid, submandibular, and sublingual salivary glands, with diverse histologic subtypes and clinical behaviors (2, 3). While major salivary gland carcinomas are uncommon, their propensity for aggressive characteristics is extensively documented, substantially contributing to the morbidity and mortality of affected patients (4). Understanding the prognostic factors and treatment outcomes of major salivary gland malignancies is crucial for optimizing patient care.

Lymph node involvement has long been recognized as a critical determinant of prognosis in head and neck cancers (5). The presence of lymph node metastasis in major salivary gland malignancies can significantly impact survival rates and guide treatment decisions. The management of nodal metastases is not controversial, with international guidelines recommending neck dissection (5, 6). But, the management of the node negative neck in patients with MSGC remains a complex and controversial issue, with various authors using different criteria to guide management of the neck, especially in those with early-stage tumors (7–9). Patients with negative cervical lymph nodes generally have a good prognosis. However, in patients without neck dissection, neck recurrences may occur after excision of the primary tumor due to occult cervical metastases (10). According to studies, the incidence of occult cervical lymph node metastasis in malignant salivary gland cancers ranges from 14.9% to 35.8%. Occult nodal metastasis has been identified as a poor prognostic factor, significantly associated with worse overall survival (11).

Therefore, the primary objective of this study is to explore the association between selective neck dissection and the survival of patients with early-stage postoperative major salivary gland malignancies. By examining a population-based cohort and adjusting for potential confounding factors, we aim to provide valuable insights into the clinical management of these relatively rare yet clinically challenging tumors.

## 2 Materials and methods

### 2.1 Data sources

The Surveillance, Epidemiology, and End Results (SEER) database (www.seer.cancer.gov) is a population-based cancer registry that captures 17 distinct population groups in 198 counties across the United States. Demographic, clinicopathological, and follow-up data for all patients with major salivary gland malignancy were extracted from the SEER*Stat 8.4.2. The study data were sourced from the publicly accessible SEER database and were provided in a de-identified format. Additionally, our research adhered to the ethical principles outlined in the 1964 Helsinki Declaration and its subsequent amendments, or equivalent ethical standards. Consequently, local ethics committee approval and institutional review board oversight were waived, and informed consents were not required.

### 2.2 Inclusion and exclusion criteria

We identified the patients diagnosed with major salivary gland malignancy using the International Classification of Disease for Oncology, 3rd Edition (ICD-O-3) topography codes for major salivary gland malignancy (C7.9, C8.0-8.1, C8.8-C8.9).

**Patients were included based on the following criteria:**

1. First malignant primary
2. Diagnosed between 2004 and 2015
3. Diagnosed with AJCC TNM stage I-II
4. Receiving definite treatment.

**Patients were excluded based on the following criteria:**

1. Unknown TNM stage information;
2. Unknown unknown survival month;
3. Surviving less than or equal to 1 month;
4. Incomplete treatment information;

### 2.3 Variables definition

Demographic and clinical variables obtained from the SEER database included age, sex, race, marital status, tumor location, tumor size, histologic grade, combined Summary Stage, AJCC (American Joint Committee on Cancer) stage, radiotherapy, chemotherapy and neck treatment strategy. Race was categorized as white, black, and other (including American Indian/Alaskan native and Asian/Pacific Islander). Marital status was categorized as married and not married. Unmarried patients included those who were never married, divorced, separated, domestic partner and widowed. Tumor location included parotid gland, submandibular gland and other Salivary (Sublingual gland, overlapping lesion of major salivary glands and others unclassified major salivary gland) in line with primary site code. Exact tumor size was measured in millimeters. Some tumors described as less than 1 cm were denoted as 9 millimeters. Less than 2 cm was recorded as 19mm. The histologic grade was categorized as well differentiated (WD), moderately differentiated (MD), poorly differentiated (PD), and undifferentiated (UD) in the SEER database. Low-grade included the former 2 groupings and high-grade included the latter 2 groupings. Combined Summary Stage divided by localized and regional. According to the AJCC TNM staging system, the early-stage patients were divided into stage I and stage II. The primary outcomes of this study were overall survival (OS) and disease-specific survival (DSS). Death from any cause was considered an event in the OS analysis, whereas death due to cancer of interest (ie, due to SGCs) was considered an event in the DSS analysis.

### 2.4 Statistical analysis

Statistical analysis was performed using the STATA software (version 15.1) and IBM SPSS Statistics (version 25). Patients were divided into 2 groups according to the neck treatment strategy, which was classified as selective neck dissection (SND) and neck observation (OBS). The Chi-square test was used to assess the differences in clinical characteristics between SND and OBS. The effects of clinicopathologic factors that influence the survival outcomes were studied through Cox proportional hazards univariate and multivariate regression analyses. Survival curves were determined using the Kaplan-Meier method and survival rates were evaluated using the log-rank test. All reported P values were 2-sided and considered statistically significant when p<0.05.

## 3 Results

### 3.1 Demographic and clinicopathologic characteristics

A total of 3778 patients were eligible for the present study. The basic characteristics are shown in **Table 1**. According to the TNM AJCC 6 staging system, 2167(57.4%) cases were of stage T1, and 1611(42.6%) were of T2 in this study. All patients had negative lymph node status. 2305 underwent SND while 1473 did not. The age of patients was categorized into two groups with a cutoff age of 50. In the whole cohort, the predominant patients were man, white race, and aged 50 or older. 82.7% of cases occurred in the parotid gland, followed by the submandibular gland (12.8%). Most cases were well differentiated or moderately differentiated. The average tumor size was 20.53 mm. Moreover, the patients who underwent SND were more inclined towards poorly differentiated (16.9% vs. 10.5%) and undifferentiated (9.9% vs. 7.2%), stage II (44.3% vs 40.0%), lager tumor size (20.89mm vs 19.95mm) and the regional combined summary stage (6.9% vs 4.2%) compared with the OBSs (p<0.05). However, there was no significant difference in distribution of race, radiotherapy and chemotherapy between the two groups. The median follow-up time was 106 months. By the end of follow-up, the OS was 77.8% for all patients, and the DSS was 92.8%. Of the patients undergoing SED, the OS was 79.4% whereas it was 75.2% for the neck observation group (p = 0.003). The DSS in the SED group was 92.7%, and it was 93.1% in the neck observation group (p = 0.580). We further analyzed the OS and DSS of patients with different levels of differentiation, and the results are presented in **Table 2**. The results showed that poorly differentiated and undifferentiated patients had poorer survival rates. We also described the survival of patients with different pathologies. The most common pathological type is Mucoepidermoid carcinoma (1165 cases), followed by Acetar cell carcinoma (825), Adenoid cystic carcinoma (452), Squamous cell carcinoma (264), Adenocarcinoma, NOS (208), Carcinoma in pleomorphic carcinoma (141), and Epithellial myoepethical carcinoma (127). Among all pathological types, Acinar cell carcinoma has better survival (OS:88.6%, DSS:96.5%), while Squamous cell carcinoma has poorer survival (OS:36.0%, DSS:80.7%). The details are shown in **Supplementary Table S1**.

**Table 1:** List of the basic information for the two cohorts.

Characteristic	Total	SND (%)	OBS (%)	p-value
	N=3778	N=2305(61.0%)	N=1473(39.0%)	
Sex, n (%)				0.035
Male	1779(47.1)	1117(48.5)	662(44.9)	
Female	1999(52.9)	1188(51.5)	811(55.1)	
Age y, n (%)				<0.001
<50	1374(36.4)	896(38.9)	478(32.5)	
≥50	2404(63.6)	1409(61.1)	995(67.5)	
Race, n (%)				0.425
White	2967(78.5)	1795(78.8)	1172(80.5)	
Black	351(9.3)	220(9.7)	131(9.0)	
Other	415(11.0)	263(11.5)	152(10.4)	
Marital status, n (%)				0.013
Married	2220(58.5)	1324(60.2)	896(64.4)	
Un-married	1370(36.3)	874(39.8)	496(35.6)	
Histologic grade, n (%)				<0.001
WD	814(21.5)	491(31.4)	323(33.8)	
MD	1116(29.5)	653(41.8)	463(48.5)	
PD	364(9.6)	264(16.9)	100(10.5)	
UD	223(5.9)	154(9.9)	69(7.2)	
Tumor location, n (%)				<0.001
Parotid gland	3124(82.7)	2018(87.5)	1106(75.1)	
Submandibular gland	484(12.8)	236(10.2)	248(16.8)	
Other Salivary	170(4.5)	51(2.2)	119(8.1)	
Tumor size, $\bar{\text{x}}$ ± s	20.53 ± 8.6	20.89 ± 8.5	19.95 ± 8.6	0.001
Combined Summary Stage, n (%)				<0.001
Localized	3556(94.1)	2145(93.1)	1411(95.8)	
Regional	222(5.9)	160(6.9)	62(4.2)	
Radiotherapy, n (%)				0.053
Yes	1664(44.0)	1044(45.3)	620(42.1)	
No	2114(56.0)	1261(54.7)	853(57.9)	
Chemotherapy, n (%)				0.535
Yes	92(2.4)	59(2.6)	33(2.2)	
No	3686(97.6)	2246(97.4)	1440(97.8)	
AJCC stage, n (%)				0.008
T1N0M0	2167(57.4)	1283(55.7)	884(60.0)	
T2N0M0	1611(42.6)	1022(44.3)	589(40.0)	
Vital status, n (%)				0.003
Dead	840(22.2)	475	365	
Alive	2938(77.8)	1830	1108	

WD, well differentiated; MD, moderately differentiated; PD, poorly differentiated; UD, undifferentiated.

**Table 2:** Survival of patients with different levels of differentiation.

Characteristic	OS (%)	p-value	DSS (%)	p-value
histologic grade		<0.001		<0.001
WD	87.1		98.2	
MD	80.1		94.2	
PD	54.1		82.7	
UD	55.6		78.5	
Combination of differentiation degree		<0.001		<0.001
low-grade	83.1		95.9	
high-grade	54.7		81.1	

### 3.2 Univariate analysis

To identify the prognostic factors associated with OS and DSS of early-stage MSGC patients, the Cox proportional hazards model was used for univariate and multivariate analyses. In univariate analysis, SND was a prognostic factor for OS in early-stage MSGC patients. Patients receiving SND showed a significant benefit in OS compared with those who were managed with neck observation [HR (95%CI): 0.849(0.741-0.973), P=0.019]. We also found statistically significant adverse outcomes in male, older-aged patients, other race, unmarried status, lager tumor sizes, regional disease stage rather than localized, lower level differentiation, tumor grades II compared to I, treatment with radiotherapy and chemotherapy (p<0.05). We did not find the associations between tumor location and OS. As for DSS, SND was not a significant prognostic factor for DSS in overall early-stage MSGC patients(p=0.549). But we found that the degree of differentiation is a significant predictor of the survival. Compared to WD patients, MD [HR (95%CI): 3.218(1.836-5.643), P<0.001], PD [HR (95%CI): 10.695(6.090-18.783), P<0.001], UD [HR (95%CI): 13.868(7.765-24.768), P<0.001] patients have a higher disease-specific risk of death. Univariate OS and DSS analysis results were in **Supplementary Table S2**.

In order to identify which patients are suitable for neck treatment, we conducted further stratified analysis. Considering the impact of differentiation on the prognosis, we divided the patients into two groups: high-grade (poorly differentiated and undifferentiated) group and low-grade (well differentiated and moderately differentiated) group. We further analyzed and found that SND is a significant predictor of OS and DSS in high-grade group other than in the low-grade group. In the high-grade patients, the survival showed a significant benefit in OS and DSS compared with the neck observations [HR for OS (95%CI): 0.571(0.446-0.731), P<0.001] and [HR for DSS (95%CI): 0.564(0.385-0.826), P=0.003]. Other factors that affect OS and DSS of patients include sex, age, tumor location and chemotherapy. Results are provided in **Table 3**.

**Table 3:** Univariate Cox proportional hazard model of OS and DSS in high-grade MSGC patients.

Variables	OS		DSS	
	HR (95% CI)	P-value	HR (95% CI)	P-value
SND				
Yes	0.571(0.446-0.731)	<0.001	0.564(0.385-0.826)	0.003
No	Reference			
Sex				
Male	1.538(1.175-2.012)	0.002	1.228(0.822-1.833)	0.316
Female	Reference			
Age				
<50	Reference			
≥50	3.846(2.351-6.292)	<0.001	1.960(1.076-3.571)	0.028
Race				
White	Reference			
Black	0.568(0.311-1.040)	0.067	0.510(0.188-1.386)	0.187
Others	0.370(0.202-0.677)	0.001	0.513(0.225-1.170)	0.112
Marital status				
Married	0.852(0.660-1.100)	0.220	0.848(0.572-1.258)	0.414
Un-married	Reference			
Combined Summary Stage				
Localized	Reference			
Regional	1.114(0.753-1.648)	0.590	1.343(0.767-2.352)	0.302
Tumor location				
Parotid gland	Reference			
Submandibular gland	1.196(0.851-1.682)	0.303	1.987(1.269-3.109)	0.003
Other Salivary	1.059(0.543-2.065)	0.867	1.130(0.414-3.084)	0.812
Tumor size	1.016(1.002-1.030)	0.028	1.016(0.995-1.039)	0.140
Radiotherapy				
Yes	0.758(0.582-0.989)	0.041	0.948(0.618-1.456)	0.808
No	Reference			
Chemotherapy				
Yes	1.366(0.891-2.096)	0.153	2.885(1.760-4.729)	<0.001
No	Reference			
AJCC stage				
T1N0M0	Reference			
T2N0M0	1.150(0.903-1.464)	0.257	1.068(0.736-1.552)	0.728

Due to differences in prognosis depending on pathology. We separate patients by histological subtype and then perform survival analysis. The results showed that the patients receiving SND treatment had better OS [HR (95%CI): 0.608(0.0.441-0.836), P=0.002] in patients with squamous cell carcinoma. But not had a significant better DSS [HR (95%CI): 0.652(0.364-1.169), P=0.151]. We conduct further hierarchical analysis, the results showed that in high-grade group, patients with squamous cell carcinoma who underwent SND had better prognosis, not only had better OS [HR (95%CI): 0.532(0.322-0.876), P=0.013], but also DSS [HR (95%CI): 0.330(0.136-0.797), P=0.014].

### 3.3 Multivariate analysis

To correct for the influence of confounding factors, we conducted a multivariate Cox proportional hazards regression analysis to verify the impact of SND on the prognosis among high-grade MSGC patients. The significant prognostic factors included in multivariate Cox proportional hazards regression analysis were SND, age, sex, race, tumor location, tumor size, radiotherapy and chemotherapy. The multivariate analysis results are presented in **Table 4**. After adjusting for the confounding variables, we found that the clinical characteristics of SND, sex, age, others races, tumor size and radiotherapy were independent predictive factors of the OS in PD or UD MSGC patients. According to the results, male, advanced age, white race compared to others races, lager tumor size, receiving chemotherapy, and neck observation were associated with poor OS. As for the DSS, the factors of SND, age, the tumor located in submandibular gland and chemotherapy were independent predictive factors of the DSS in high-grade MSGC patients. Above all, the multivariate analysis revealed that, compared with neck observation, therapeutic neck dissection resulted in a significant independent OS [HR 95%CI: 0.604(0.470-0.776), P<0.001] and DSS [HR 95% CI: 0.630(0.426-0.932), P=0.021] advantage in patients with high-grade MSGC. Additionally, it was worth mentioning that age was an important risk prognostic factor both in OS and DSS. Therefore, in elderly patients, special attention should be paid to treatment strategies and follow-up.

**Table 4:** Multivariate Cox proportional hazard model of OS and DSS in high-grade MSGC patients.

Variables	OS		DSS	
	HR (95% CI)	P-value	HR (95% CI)	P-value
SND				
Yes	0.604(0.470-0.776)	<0.001	0.630(0.426-0.932)	0.021
No	Reference			
Sex				
Male	1.433(1.090-1.885)	0.010	1.163(0.775-1.747)	0.466
Female	Reference			
Age				
<50	Reference			
≥50	3.535(2.151-5.809)	<0.001	2.030(1.101-3.741)	0.023
Race				
White	Reference			
Black	0.632(0.344-1.159)	0.138	0.560(0.205-1.532)	0.259
Others	0.375(0.203-0.694)	0.002	0.433(0.186-1.004)	0.051
Tumor location				
Parotid gland	Reference			
Submandibular gland	1.481(1.044-2.102)	0.208	2.364(1.486-3.761)	<0.001
Other Salivary	1.306(0.667-2.560)	0.436	1.176(0.427-3.241)	0.754
Tumor size	1.019(1.005-1.035)	0.009	1.019(0.997-1.042)	0.087
Radiotherapy				
Yes	0.702(0.536-0.920)	0.010	0.822(0.530-1.275)	0.381
No	Reference			
Chemotherapy				
Yes	1.450(0.936-2.245)	0.096	3.094(1.851-5.173)	<0.001
No	Reference			

Then, we also conducted multivariate Cox proportional hazard regression analysis to verify whether the high-grade squamous cell carcinoma MSGC patients has independent better survival after receiving SND. After adjusting for confounding factors, compared with neck observation, receiving neck dissection resulted in a significant independent OS (HR, 0.555; 95% CI, 0.328-0.941; P=0.029) and DSS (HR, 0.349; 95% CI, 0.142-0.858; P=0.022) advantage in patients with high-grade squamous cell carcinoma of MSGC. And after adjustment, the impact of confounding factors (including age, sex, race, tumor location, tumor size, radiotherapy and chemotherapy) on the prognosis of patients was not statistically significant.

**Figure 1:**
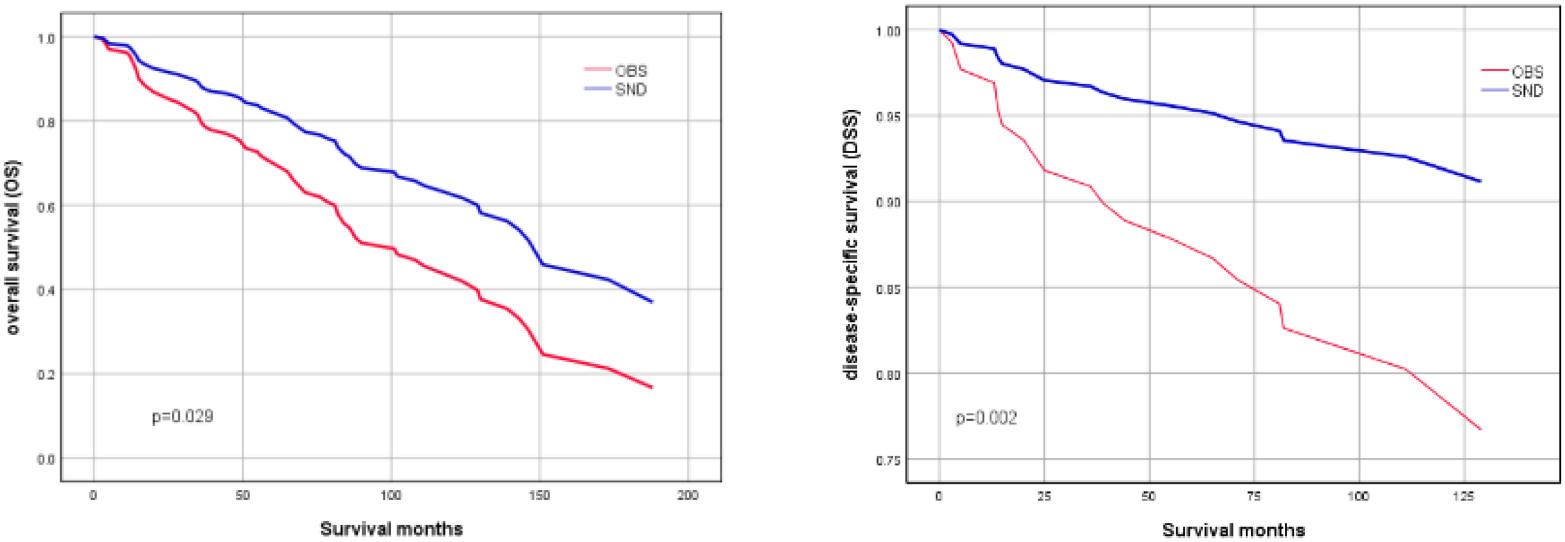
OS (left image) and DSS (right image) of patients with high-grade squamous cell carcinoma of MSGC, stratified by SND and OBS. (Adjusted by age, sex, race, tumor location, tumor size, radiotherapy and chemotherapy).

To further investigate the effects of SND on OS and DSS, Kaplan-Meier survival curves were plotted according to the neck treatment policies. The survival curves demonstrated that adjusted SND still had significantly better OS(P=0.029) and DSS(P=0.022) than the observation group in patients with high-grade squamous cell carcinoma of MSGC (**Figure 1**).

## 4 Discussion

Established guidelines, or their absence, have added complexity to the evaluation of salivary gland tumors in different medical centers. Consensus within the current literature delineates strategies for handling clinically evident nodal involvement, which predominantly involves surgical intervention, specifically selective neck dissection, often followed by postoperative radiotherapy (12). However, the management of the clinically N0 neck in individuals diagnosed with salivary gland carcinoma remains a topic of ongoing debate and uncertainty. Nevertheless, elective management in the cN0 neck was warranted (13). Certain experts advocate performing neck dissections exclusively for individuals displaying clinical signs of nodal disease, while others suggest this procedure for patients with adverse prognostic indicators such as high histopathological grade, locally advanced (stage T3-T4) (13–17). A wait-and-see approach is justified in patients with a probability of occult metastases <19% (8). There is no consensus on how to choose between wait and see or neck dissection in early salivary gland patients. There is a fundamental need for additional studies on the prognosis after neck dissection to better guide clinical decision-making for patients with these rare tumors and clinically negative necks especially in the early stages.

In this study, we used SEER data sets to compare the outcomes of different treatment policies for N0 neck in early-stage MSGC. On the whole, patients who underwent neck dissection demonstrated a significant survival benefit in OS than those who were managed with neck observation only, whether in univariate regression or multivariate regression analysis. As for the DSS, in the overall population, the patients still have the trend to benefit from SND, although the result did not meet the significance level. This also means that in the overall patients, some patients can benefit from SND, yet some people cannot. In order to prove and identify patients who can benefit more from SND treatment, we conducted a further stratified analysis. Considering the significant impact of disease differentiation on patient prognosis, we made a hierarchical analysis of the differentiation. We found that high-grade patients can get better survival benefit after receiving SND. These results fully illustrate the important clinical value of SND in the prognosis of high-grade early stage MSCG.

Can patients with all the pathological conditions benefit from SND? We conducted further analysis to identify pathological subtype that are more likely to benefit from SND. We observed that patients with squamous cell carcinoma stood out, and notably, the high-grade squamous cell carcinoma patients undergoing SND showed significant advantages in both OS and DSS. In the multivariable regression analysis, after adjusting for confounding factors, these results still maintain statistical significance. These results indicated that SND is necessary for high-grade early-stage MSGC patients, especially in the key pathological subgroup, even if there is no evidence of cervical metastasis. This phenomenon can be explained from the following aspects. high-grade MSGC are more often associated with the risk of occult neck metastasis than low-grade tumors (18–20). In addition, histology of MSGC plays a major role. Some histological types have shown a greater potential to develop occult nodal metastasis, and squamous cell carcinoma is included (21). The treatment of the cN0 neck aims to eradicate any existing microscopic lymph node disease, in patients with a high risk of occult lymph node metastatic disease. This explains the reason why SND can achieve better survival in this part of the population. In our study, regional node positive detection was performed, but the results were all negative. However, this cannot rule out the possibility of occult metastasis in undetected lymph nodes. Because the results of regional nodes positive detection in our data are all negative, we did not describe the incidence rate of occult metastasis, which is a limitation of this study. But we believe that this does not affect the reliability of the results. In terms of radiotherapy, we found an interesting phenomenon that radiotherapy yielded survival benefits for high-grade patients, and this is consistent with existing studies (22, 23). However, the influence of confounding factors including radiotherapy was adjusted in our multivariate analysis, and adjusted results showed that SND still had a significant survival advantage in the target population.

Although there have been similar studies on this topic, they mainly revolve around lymph node metastasis (15). Several factors strengthen our study conclusions. First, our results are based on the analysis of a large cohort of 3778 patients. All relevant data were retrieved from the SEER database, which contains population-based data on overall and disease-specific survival. The results may be generalized to a wider population of patients. Second, we incorporated a comprehensive range of factors into the Cox proportional hazards regression model for multivariate analysis. Third, in our study, we conduct a stratified analysis according to a wide range of prognostic factors. These results will provide clinicians with valuable information to assist them in selecting the most appropriate treatment policy for patients with early-stage MSGC. About the security of SND, a study by Brauer et al. was conducted to evaluate the safety of SND, concluding that although there were more post-operative complications, there was no significant impact on the rate of readmissions of reoperations (18).

In general, selective neck lymph node dissection can improve the survival in early-stage major salivary gland carcinoma in the key groups. Special attention should be paid to the lymph node status of early patients during preoperative examination. Imaging examinations and ultrasound examinations have important value in distinguishing between benign and malignant lymph nodes, and developing new technologies to improve the detection rate of occult lymph node metastasis is of great significance.

Our study has several limitations common to observational studies. First, the heterogeneity between institutions of the SEER database may result in diagnostic and therapeutic discrepancies such as the pathologic analysis of specimens or surgical technique. Second, our analysis lacks data regarding postoperative neck lymph node metastasis, which could help differentiate the beneficiaries of neck dissection and predict patients’ prognosis. Third, we were unable to access information regarding the implementation of postoperative surveillance in patients who underwent neck observation. For example, details regarding the frequency of postoperative neck examinations were unavailable. Fourth, the SEER database lacks records of radiotherapy dose and duration and chemotherapy cycle, which may also influence the survival. The SEER database is population-based, and these limitations are unlikely to affect our main conclusions, but more studies will be needed in the future to further validate our findings. Despite these limitations, our study provides insights into the debatable issue of managing the node-negative neck in MSGC.

## 5 Conclusion

In summary, this extensive study, encompassing a substantial cohort of T1/T2N0M0 major salivary gland carcinoma (MSGC) cases, has meticulously examined the effectiveness of selective neck dissection (SND) compared to a strategy of neck observation. Our findings robustly demonstrate that SND significantly enhances the overall and disease-specific survival rates in early-stage MSGC patients with poorly differentiated or undifferentiated. Especially in the pathological state of squamous cell carcinoma. These conclusive findings should serve as a catalyst for future research endeavors, guiding the exploration of optimal neck management strategies tailored for early-stage major salivary gland carcinoma patients.

## Data availability statement

The datasets presented in this study can be found in online repositories. The names of the repository/repositories and accession number(s) can be found in the article/**Supplementary Material**.

## Ethics statement

The studies involving humans were approved by the ethical principles outlined in the 1964 Helsinki Declaration and its subsequent amendments, or equivalent ethical standards. The studies were conducted in accordance with the local legislation and institutional requirements. Written informed consent for participation was not required from the participants or the participants’ legal guardians/next of kin in accordance with the national legislation and institutional requirements. The studies were conducted in accordance with the local legislation and institutional requirements. Written informed consent for participation was not required from the participants or the participants’ legal guardians/next of kin in accordance with the national legislation and institutional requirements. Written informed consent was not obtained from the individual(s) for the publication of any potentially identifiable images or data included in this article because The study data were sourced from the publicly accessible SEER database and were provided in a de-identified format. Our research adhered to the ethical principles outlined in the 1964 Helsinki Declaration and its subsequent amendments, or equivalent ethical standards. Consequently, local ethics committee approval and institutional review board oversight were waived, and informed consents were not required.

## Author contributions

MA: Conceptualization, Software, Writing – original draft, Data curation, Formal analysis, Writing – review & editing. JZ: Investigation, Methodology, Writing – review & editing. FY: Conceptualization, Writing – review & editing. PX: Conceptualization, Data curation, Funding acquisition, Project administration, Supervision, Writing – review & editing.

## References

[1] van Herpen C Vander Poorten V Skalova A Terhaard C Maroldi R van Engen A. et al: Salivary gland cancer: ESMO–European Reference Network on Rare Adult Solid Cancers (EURACAN) Clinical Practice Guideline for diagnosis, treatment and follow-up. ESMO Open. (2022) 7(6):100602. doi: 10.1016/j.esmoop.2022.100602 36567082 PMC9808465

[2] Eveson JW Cawson RA. Salivary gland tumours. A review of 2410 cases with particular reference to histological types, site, age and sex distribution. J Pathol. (1985) 146:51–8. doi: 10.1002/path.1711460106 4009321

[3] Fonseca FP Carvalho Mde V de Almeida OP Rangel AL Takizawa MC Bueno AG. Clinicopathologic analysis of 493 cases of salivary gland tumors in a Southern Brazilian population. Oral Surg Oral Med Oral Pathol Oral Radiol. (2012) 114:230–9. doi: 10.1016/j.oooo.2012.04.008 22769409

[4] Gatta G Guzzo M Locati LD McGurk M Prott FJ. Major and minor salivary gland tumours. Crit Rev Oncol Hematol. (2020) 152:102959. doi: 10.1016/j.critrevonc.2020.102959 32485526

[5] Lombardi D Tomasoni M Paderno A Mattavelli D Ferrari M Battocchio S. et al: The impact of nodal status in major salivary gland carcinoma: A multicenter experience and proposal of a novel N-classification. Oral Oncol. (2021) 112:105076. doi: 10.1016/j.oraloncology.2020.105076 33137587

[6] Sood S McGurk M Vaz F. Management of salivary gland tumours: United Kingdom national multidisciplinary guidelines. J Laryngol Otol. (2016) 130:S142–9. doi: 10.1017/S0022215116000566 PMC4873929 27841127

[7] Nobis C-P Rohleder NH Wolff K-D Wagenpfeil S Scherer EQ Kesting MR. Head and neck salivary gland carcinomas—Elective neck dissection, yes or no? J Oral Maxillofac Surg. (2014) 72:205–10. doi: 10.1016/j.joms.2013.05.024 23891016

[8] Wong WK Morton RP. Elective management of cervical and parotid lymph nodes in stage N0 cutaneous squamous cell carcinoma of the head and neck: a decision analysis. Eur Arch Oto-Rhino-Laryngol. (2013) 271:3011–9. doi: 10.1007/s00405-013-2857-6 24337900

[9] Chen AM Garcia J Lee NY Bucci MK Eisele DW. Patterns of nodal relapse after surgery and postoperative radiation therapy for carcinomas of the major and minor salivary glands: What is the role of elective neck irradiation? Int J Radiat OncolBiolPhysics. (2007) 67:988–94. doi: 10.1016/j.ijrobp.2006.10.044 17234357

[10] Valstar MH van den Brekel MWM Smeele LE. Interpretation of treatment outcome in the clinically node-negative neck in primary parotid carcinoma: A systematic review of the literature. Head Neck. (2009) 32:1402–11. doi: 10.1002/hed.21316 20029981

[11] Tranchito E Cabrera C Terry M Li S Thuener JE Fowler N. Occult nodal metastasis in major salivary gland Malignancy: An update from the National Cancer Database. Oral Oncol. (2022) 128:105829. doi: 10.1016/j.oraloncology.2022.105829 35349935

[12] van Herpen C Vander Poorten V Skalova A Terhaard C Maroldi R van Engen A. et al: Salivary gland cancer: ESMO-European Reference Network on Rare Adult Solid Cancers (EURACAN) Clinical Practice Guideline for diagnosis, treatment and follow-up. ESMO Open. (2022) 7:100602. doi: 10.1016/j.esmoop.2022.100602 36567082 PMC9808465

[13] Ng-Cheng-Hin B Glaholm J Awad Z Gujral DM. Elective management of the neck in parotid tumours. Clin Oncol. (2018) 30:764–72. doi: 10.1016/j.clon.2018.08.017 30220613

[14] Yoo SH Roh JL Kim SO Cho KJ Choi SH Nam SY. Patterns and treatment of neck metastases in patients with salivary gland cancers. J Surg Oncol. (2015) 111:1000–6. doi: 10.1002/jso.23914 25976866

[15] Ketterer MC Konrad Dahlem KK Häussler SM Jakob TF Pfeiffer J Becker C. Clinical significance and indication for surgical treatment of occult cervical and intraglandular nodal involvement in parotid Malignancy. J Oral Maxillofac Surg. (2019) 77:2355–61. doi: 10.1016/j.joms.2019.04.009 31077673

[16] Feinstein TM Lai SY Lenzner D Gooding W Ferris RL Grandis JR. Prognostic factors in patients with high-risk locally advanced salivary gland cancers treated with surgery and postoperative radiotherapy. Head Neck. (2011) 33:318–23. doi: 10.1002/hed.21444 PMC4135528 21284048

[17] Fussey J Tomasoni M Tirelli G Giordano L Galli A Colangeli R. et al: Prognostic indicators in clinically node-negative Malignant primary salivary tumours of the parotid: A multicentre experience. Oral Oncol. (2021) 123:105577. doi: 10.1016/j.oraloncology.2021.105577 34742011

[18] Gilbert MR Sharma A Schmitt NC Johnson JT Ferris RL Duvvuri U. A 20-year review of 75 cases of salivary duct carcinoma. JAMA Otolaryngol–Head Neck Surg. (2016) 142(5):489–95. doi: 10.1001/jamaoto.2015.3930 PMC5033043 26939990

[19] Seethala RR. An update on grading of salivary gland carcinomas. Head Neck Pathol. (2009) 3:69–77. doi: 10.1007/s12105-009-0102-9 20596994 PMC2807532

[20] Armstrong JG Harrison LB Thaler HT Friedlander-Klar H Fass DE Zelefsky MJ. The indications for elective treatment of the neck in cancer of the major salivary glands. Cancer. (1992) 69:615–9. doi: 10.1002/1097-0142(19920201)69:3<615::AID-CNCR2820690303>3.0.CO;2-9 1730113

[21] Vedula S Shah YS Barinsky GL. Utility of elective neck dissection in clinically node-negative parotid Malignancy. Otolaryngol Head Neck Surg. (2023) 169:917–27. doi: 10.1002/ohn.264 36807904

[22] Park JB Wu HG Kim JH Lee JH Ahn SH Chung EJ. Adjuvant radiotherapy in node-negative salivary Malignancies of the parotid gland: A multi-institutional analysis. Radiother Oncol. (2023) 183:109554. doi: 10.1016/j.radonc.2023.109554 36813174

[23] Morand GB Eskander A Fu R de Almeida J Goldstein D Noroozi H. The protective role of postoperative radiation therapy in low and intermediate grade major salivary gland Malignancies: A study of the Canadian Head and Neck Collaborative Research Initiative. Cancer. (2023) 129:3263–74. doi: 10.1002/cncr.34932 37401841

