# Supplementary material for: The prognostic value of selective neck dissection in early-stage major salivary gland carcinoma: a population-based analysis
Source: Front Oncol. 2024 May 22;14:1347339. doi: 10.3389/fonc.2024.1347339 (PMC11150835; doi:10.3389/fonc.2024.1347339)
Supplement: Supplementary file 1 [file Table_1.docx]

Table S1: Survival rate of patients with different pathologies

| Characteristic | N | OS (%) | DSS (%) |
| --- | --- | --- | --- |
| Mucoepidermoid carcinoma | 1165 | 84.5 | 96.7 |
| Acinar cell carcinoma | 825 | 88.6 | 96.5 |
| Adenoid cystic carcinoma | 452 | 78.3 | 88.5 |
| Squamous cell carcinoma, NOS | 264 | 36.0 | 80.7 |
| Adenocarcinoma, NOS | 208 | 68.3 | 88.9 |
| Carcinoma in pleomorphic adenoma | 141 | 83.0 | 93.6 |
| Epithelial-myoepithelial carcinoma | 127 | 78.0 | 94.5 |
| Basal cell adenocarcinoma | 87 | 77.0 | 94.3 |
| Malignant myoepithelioma | 64 | 75.0 | 92.2 |
| Pleomorphic carcinoma | 62 | 77.4 | 95.2 |
| Infiltrating duct carcinoma, NOS | 48 | 64.6 | 83.3 |
| Secretory carcinoma of breast | 35 | 85.7 | 100 |
| Lymphoepithelial carcinoma | 20 | 85.0 | 100 |
| Carcinoma, NOS | 104 | 60.6 | 85.6 |
| others | 176 | 63.6 | 85.8 |
