# Supplementary material for: The prognostic value of selective neck dissection in early-stage major salivary gland carcinoma: a population-based analysis
Source: Front Oncol. 2024 May 22;14:1347339. doi: 10.3389/fonc.2024.1347339 (PMC11150835; doi:10.3389/fonc.2024.1347339)
Supplement: Supplementary file 2 [file Table_2.docx]

Table S2 Univariate Cox proportional hazard model of MSGC OS and DSS .

| Variables | OS | | DSS | |
| --- | --- | --- | --- | --- |
|  | HR (95% CI) | P-value | HR (95% CI) | P-value |
| SND |  |  |  |  |
| Yes | 0.849(0.741-0.973) | 0.019 | 1.078(0.843-1.380) | 0.549 |
| No | Reference |  |  |  |
| Sex |  |  |  |  |
| Male | 1.833(1.597-2.104) | ＜0.001 | 1.735(1.362-2.212) | ＜0.001 |
| Female | Reference |  |  |  |
| Age |  |  |  |  |
| ＜50 | Reference |  |  |  |
| ≥50 | 8.106(6.292-10.443) | ＜0.001 | 4.493(3.135-6.439) | ＜0.001 |
| Race |  |  |  |  |
| White | Reference |  |  |  |
| Black | 0.789(0.617-1.008) | 0.789 | 0.709(0.449-1.119) | 0.140 |
| Others | 0.449(0.335-0.601) | ＜0.001 | 0.561(0.352-0.896) | 0.016 |
| Marital status |  |  |  |  |
| Married | 0.857(0.744-0.987) | 0.032 | 1.053(0.815-1.360) | 0.694 |
| Un-married | Reference |  |  |  |
| Histologic grade |  |  |  |  |
| WD | Reference |  |  |  |
| MD | 1.554(1.232-1.960) | ＜0.001 | 3.218(1.836-5.643) | ＜0.001 |
| PD | 4.080(3.196-5.209) | ＜0.001 | 10.695(6.090-18.783) | ＜0.001 |
| UD | 4.219(3.206-5.553) | ＜0.001 | 13.868(7.765-24.768) | ＜0.001 |
| Combined Summary Stage |  |  |  |  |
| Localized | Reference |  |  |  |
| Regional | 1.654(1.295-2.112) | ＜0.001 | 2.544(1.776-3.646) | ＜0.001 |
| Tumor location |  |  |  |  |
| Parotid gland | Reference |  |  |  |
| Submandibular gland | 1.150(0.949-1.393) | 0.155 | 1.620(1.195-2.196) | 0.002 |
| OthSalivary | 0.987(0.709-1.374) | 0.937 | 1.144(0.653-2.004) | 0.638 |
| Tumor size | 1.035(1.027-1.043) | ＜0.001 | 1.052(1.038-1.066) | ＜0.001 |
| Radiation therapy |  |  |  |  |
| Yes | 1.399(1.222-1.602) | ＜0.001 | 2.221(1.734-2.844) | ＜0.001 |
| No | Reference |  |  |  |
| Chemotherapy |  |  |  |  |
| Yes | 2.669(1.963-3.627) | ＜0.001 | 5.991(4.098-8.759) | ＜0.001 |
| No | Reference |  |  |  |
| AJCC stage |  |  |  |  |
| T1N0M0 | Reference |  |  |  |
| T2N0M0 | 1.620(1.414-1.855) | ＜0.001 | 1.915(1.505-2.436) | ＜0.001 |
